# Bacterial Communities in Aerosols and Manure Samples from Two Different Dairies in Central and Sonoma Valleys of California

**DOI:** 10.1371/journal.pone.0017281

**Published:** 2011-02-18

**Authors:** Subbarao V. Ravva, Chester Z. Sarreal, Robert E. Mandrell

**Affiliations:** Produce Safety and Microbiology Research Unit, Western Regional Research Center, Agriculture Research Service, United States Department of Agriculture, Albany, California, United States of America; Argonne National Laboratory, United States of America

## Abstract

Aerosols have been suspected to transport food pathogens and contaminate fruits and vegetables grown in close proximity to concentrated animal feeding operations, but studies are lacking that substantiate such transport. To monitor the potential transport of bacteria originated from fresh or dry manure through aerosols on a dairy, we identified by 16S rRNA sequencing, bacteria in aerosols collected within 2 to 3 meters from dairy cows at two dairies. Gram-positive *Firmicutes* were predominant in aerosols from a dairy in Sonoma, California, and surrounded by vineyards, in contrast to sequences of Gram-negative *Proteobacteria* predominant in aerosols from a dairy in Modesto, California, also surrounded by other dairies. Although *Firmicutes* represented approximately 50% of the 10 most abundant sequences, aerosols from the Sonoma dairy also contained sequences of *Bacteriodetes* and *Actinobacteria*, identified previously with animal feces. While none of the top 10 sequences from fresh or dry manure from Modesto dairy were detected in aerosols, two of the sequences from the phylum *Bacteriodetes* and one from class *Clostridia* from fresh manure were detected in aerosols from Sonoma. Interestingly, none of the sequences from dry manure were in the top 10 sequences in aerosols from both dairies. The 10 most abundant sequences in aerosols from the Modesto dairy were all from *Proteobacteria* and nearly half of them were from genus *Massilia*, which have been isolated previously from immune-compromised people and aerosols. We conclude that the predominant bacteria in aerosols are diverse among locations and that they do not reflect the predominant species of bacteria present in cow feces and/or in close proximity to cows. These results suggest that the aerosol sequences did not originate from manure. Large volumes of aerosols would be required to determine if bacterial sequences from aerosols could be used to track bacteria in manure to crops grown in proximity.

## Introduction

Aerosols have been suspected to transport food pathogens and contaminate fruits and vegetables grown in close proximity to animal raising operations, but studies are lacking that identify the mechanisms of transport. Concentrated animal feeding operations (CAFO) produce large amounts of waste [1], thus raising concerns of pathogen transport through aerosols generated by waste processing on-site. The California Central Valley has locations with a high density of CAFOs and this region experiences occasional high winds and dust storms, thus creating the potential for pathogen transmission through aerosols. A mid-size dairy of 1000 cows will produce more than 12,000,000 kg of manure per year [2] and the manure is usually stored/processed on-site. This increases the risk of transporting bacterial pathogens by a number of mechanisms (water, wildlife, dust) and is of special concern where fruit and vegetable crops are grown nearby. Enteric bacterial pathogens causing much of the foodborne illnesses, including Salmonellae, *Campylobacter* sp., *Listeria monocytogenes*, and *Escherichia coli* O157:H7, have been reported to survive for long periods in manure and manure slurries [3], [4] and a potential exists for their transmission through aerosols.

Aerosol transmission of *E. coli* O157:H7 was observed in one study with pigs penned 3 to 6 m away from pigs inoculated experimentally in an enclosed room [5], but transmission was not detected in sheep in a similar experiment. In a recent field study, a marked strain of *E. coli* K12 was detected 125 m downwind from a rain gun spray application of *E. coli-*treated pig slurry to grass pasture [6]. Aerosol transport of *Salmonella* through contaminated dust to turkeys in a simulated holding-shed environment [7], and significant bacterial population changes in aerosols in poultry houses from pre-flock to late-flock stages, were observed by 16s rRNA analyses [8].

The viable microflora present in aerosols where food crops are grown are relevant, especially in regions where there is a high density of cattle nearby. Nevertheless, field studies of aerosols from dairy and agricultural production areas are lacking. Thus, we characterized and compared bacterial communities in aerosols collected in close proximity to dairy cows to those present in fresh and dry manure collected on-site. We compared bacterial community diversity by sampling on two different dairies selected for different manure management practices and land usage in the surrounding region (dairies vs. vineyards). We used high volume cyclonic air samplers to collect aerosols and measured the bacterial communities by16S rRNA gene sequencing. Aerobic bacteria were cultured from aerosols also to compare to the 16S community structure.

## Materials and Methods

### Ethics statement

Air and manure samples were collected without unduly disturbing the dairy cows or interrupting any normal animal raising operations followed on the farms.

### Bioaerosol and manure samples from dairies

**Figure 1:**
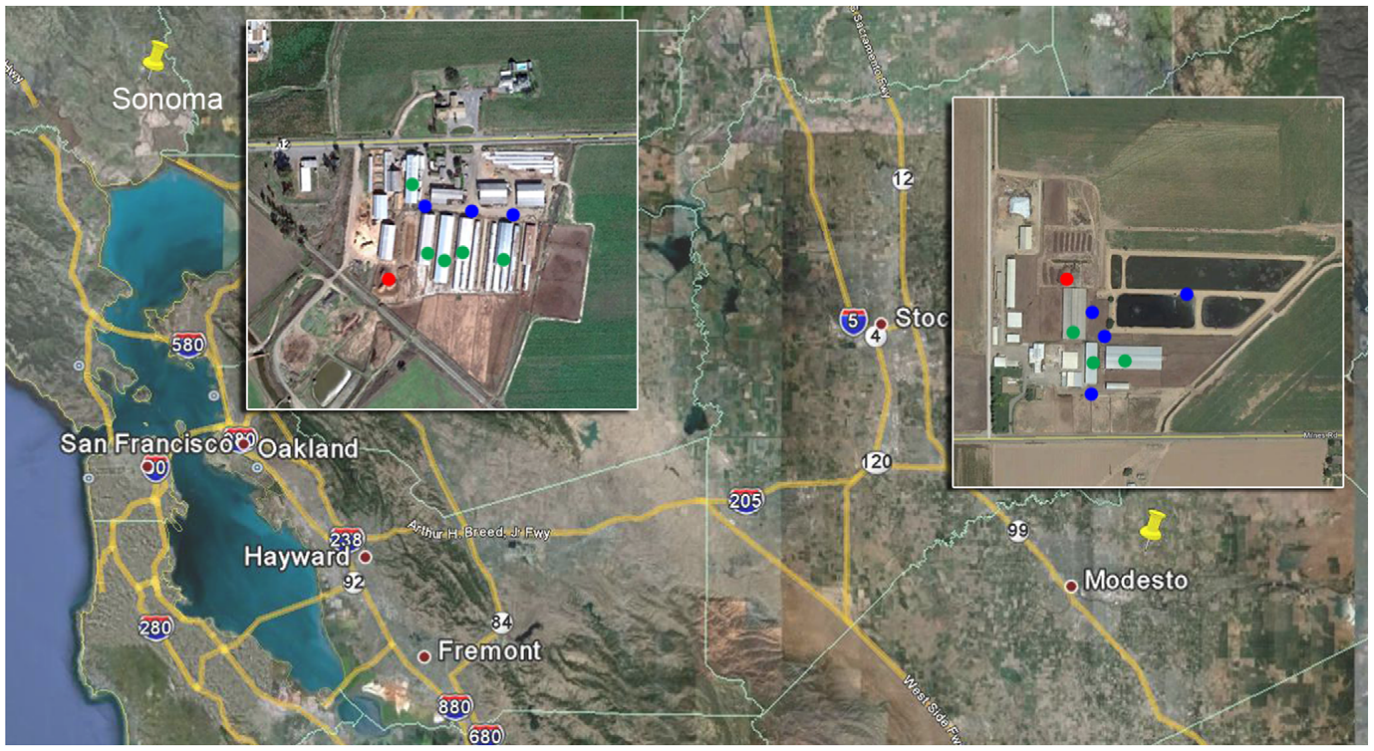
Google map showing the locations of dairies in California and the sampling locations. Blue dots are aerosol sampling locations. Green dots represent the stalls from where fresh manure was collected. Red dots are on-site locations for dry manure. The same color coding is used in Figures 2, 3 and 5 to designate the sample sources for the bacterial taxa.

Aerosols, fresh and dry manure samples (Figure 1) were collected from two medium-sized dairies (*ca*. 800 to 1000 milking cows) located in San Joaquin valley near Modesto, CA and a location near Sonoma, CA. The two dairies are separated by 190 km and were sampled at various intervals during March to September of 2006. The Modesto dairy is located centrally amongst numerous other dairies and orchards; the Sonoma dairy is an isolated dairy surrounded by vineyards. The Modesto dairy has an on-site manure solid-liquid separator and the liquid is collected in two holding lagoons (*c.*10^8^ liters) equipped with circulating aerators whereas the Sonoma dairy did not use a solid separator or circulators in the lagoon. Dust storms that transport particulate manure are quite frequent in the San Joaquin valley (i.e., Modesto dairy) and historic records indicate wind gusts of 60 to 70 kph have occurred at both locations. Average recorded annual rainfall was 36 cm for Modesto and 60 cm for Sonoma valley.

Aerosols were collected using a portable multi-stage wetted-wall air sampler (SASS 2000 Plus, Research International, Monroe, WA) with an air flow of 270 L/min. The units were placed on stands at 1 m above ground level and 2 to 3 m away from the dairy stalls to minimize the collection of heavy dust particles in the aerosol wash chamber and to maximize the collection of fine particles distributed in the air by cattle movement in the barns. Temperature, wind speed and relative humidity were monitored using a Kestrel 4000 Pocket Weather Tracker (Nielsen-Kellerman, Boothwyn, PA). Wind direction at the time of aerosol samplings at the Sonoma dairy was SSE 139° to 148° and SSW 200°, whereas it was NNE 13° to 26° at the Modesto dairy.

Airborne particles were collected using 5 mL sterile water to wash incoming air, aerosols were collected into a sterile 50 mL tube every 3 min with a 2 min transfer-refill cycle for a total of 30 min for each unit. Four samplers were placed at each location to yield a total collection time of 2 h per location. Samplings were done at 4 locations on the Modesto dairy and 3 locations on the Sonoma dairy (Figure 1) and the aerosol wash-water was pooled from all units and locations for characterization of aerobic bacteria and extraction of environmental DNA.

Fresh manure was collected using a sterile spatula from freshly dropped feces and combined from 5 cows; a total of 5 samples were created this way. For sequencing purposes, the 5 fresh manure samples were combined. Dried manure samples were collected from manure piles located on-site. Both manure and aerosol samples were stored on ice and transported to the laboratory where aerosol samples were processed on the same day for enumeration of aerobic bacteria. Remaining aerosol wash-water and manure samples were stored at −80°C until used for extraction of genomic DNA.

Aerosol wash-water, without further concentration, was used for the enumeration of aerobic bacteria using Petrifilm count plates (3 M, St. Paul, MN) [9]. One-milliliter portions of 10-fold serial dilutions of wash-water in phosphate buffered saline (0.01 M, pH 7.4) were plated and incubated at 25°C for 2 days.

### Extraction of DNA

Aerosol wash-waters were filtered through 0.2 µm cellulose membrane filters (13 mm Millipore, Billerica, MA). Filters with aerosol particles were pooled and treated with 200 µL DNAzol Direct (Molecular Research Center, Inc, Cincinnati, OH) in 1.5 mL centrifuge tubes and heated to 95°C for 20 min. Tubes were centrifuged at 10,000×*g* for 5 min and 100 µL of the supernatant containing DNA was used without any further purification for amplification of 16S rRNA.

Duplicate half-gram manure pellets (both fresh and dry) were extracted using the alternate protocol of the MoBio UltraClean soil DNA isolation kit (MoBio, Solano Beach, CA) and the wash steps designated 15 to 21 in the protocol were repeated to remove a “green tinge” from DNA extract.

DNA was quantified by the Quanti-iT Picogreen dsDNA assay (Invitrogen Corporation, Carlsbad, CA). Purified DNA from aerosol and manure samples was suspended at a concentration of 1 to 5 ng/µL in UltraClean PCR water.

### Amplification of 16S rRNA

A 236-bp fragment of the 16S rRNA gene was amplified using eubacterial primers PRBA338f (5′ ACTCCTACGGGAGGCAGCAG 3′) and PRUN518r (5′ ATTACCGCGGCTGCTGG 3′) [10]. Briefly, genomic DNA from environmental samples was amplified in 10 replicates of 50-µL reaction mixtures containing 5 to 50 ng of template DNA, 300 nM of each primer and two Illustra PuReTaq Ready-To-Go PCR Beads (GE Healthcare, Piscataway, NJ). PCR reactions were performed using DNA engine DYAD® Peltier Thermal Cycler (Bio-Rad Labs., Hercules, CA) using the protocol: one cycle of 92°C for 2 min; 30 cycles of 92°C for 1 min, 55°C for 30 s, and 72°C for 1 min; and one cycle of 72°C for 15 min. PCR products were pooled from all 10 reactions and purified with DNA Clean and Concentrator-5 kit (Zymo Research, Orange, CA) by using 5 volumes of DNA binding buffer to each volume of PCR product mixture as per manufacture's instructions.

### 16S rRNA clone libraries

PCR products were cloned using TOPO TA cloning kit as per manufacture's instructions and transformed into *E. coli* TOP10F' One Shot competent cells (Invitrogen, Carlsbad, CA). Clones were plated on LB agar plates containing kanamycin (50 µg/mL), isoprophyl-β-D-thiogalactopyranoside (IPTG, 20 mM), and 5-bromo-4-chloro-3-indolyl-β-D-galactopyranoside (X-gal, 80 µg/mL). Two PCR reactions were performed for each sample, and 96 clones were picked from each PCR to minimize potential PCR bias. Procedures for preparation of DNA templates using the Illustra Templiphi 100 amplification kit (GE Healthcare), sequencing reactions using the Big Dye Terminator v3.1 cycle sequencing kit (Applied Biosystems, Foster City, CA), cleaning sequencing reactions with DyeEx 96 kit (Qiagen, Valencia, CA) and electrophoresis with an Applied Biosystems 3730 DNA analyzer were same as described earlier [11], except that we used the reverse primer PRUN518r for unidirectional sequencing reactions.

### DNA sequence analysis and dendrogram construction

DNA sequences were analyzed manually for bases called incorrectly, then trimmed at both 5′ and 3′ ends using Kodon (v.3.5, Applied Maths, Inc., Austin, TX). Only sequences with unambiguous reads were compiled into data sets and were aligned with the closest sequence relatives from GenBank database by using Kodon. Phylogenetic trees of the 16S rRNA sequences were constructed by using a neighbor-joining method incorporating Jukes-Cantor distance correction. Tree stability was assessed by bootstrap analysis with 1,000 iterations. The phylogenetic relationships between the characterized sequences were analyzed by Bionumerics (v.6.5, Applied Maths) to identify closely related genotypes. Taxonomic assignment of 16S rRNA sequences was performed using the RDP Naïve Bayesian rRNA Classifier Version 2.2 of the Ribosomal Database Project [12].

### Diversity indices and library comparisons

Rarefaction curves of clone libraries from each sample were generated using operational taxonomic units (OTUs) with >97% 16S rRNA sequence similarities with GenBank accessions. Rarefaction analysis with 95% confidence levels was performed using aRarefactWin by Holland (Analytic Rarefaction v.1.3; S. Holland, Stratigraphy Lab, University of Georgia, Athens; http://uga.edu/strata/software/Software.html). Species richness and diversity were determined for each library of OTUs using Simpson's and Shannon-Wiener index of diversities (Bionumerics). Library Compare tool (http://rdp.cme.msu.edu/comparison/comp.jsp) of the Ribosomal Database Project [12] was used to determine if the library of OTUs from aerosols differ significantly at any hierarchial taxon level from OTU libraries created from fresh or dry manure collected from the same dairy (Figure S1). OTU libraries created from aerosols from both dairies were also compared (Figure S1).

### Nucleotide sequence accession numbers

Representative DNA sequences of the top 10 bacterial sequences from aerosols, fresh and dry manure from both dairies were submitted to GenBank under accession numbers HQ340004 to HQ340063. The GenBank accession numbers corresponded to sequences S1 to S30 for sequences originated from the Sonoma dairy and M1 to M30 for sequences from the Modesto dairy.

## Results

### Bacterial communities in aerosol and manure samples from Sonoma dairy

A total of 495 clones representing 6 different bacterial phyla were characterized from aerosol, fresh and dry manure samples collected from the Sonoma dairy (Table 1). Sequences of Gram-positive *Firmicutes* predominated in aerosol and fresh manure samples and represented more than half of all sequences, compared to Gram-negative *Proteobacteria* predominating in the bacterial communities in dry manure. Gram-negative *Bacteriodetes* were the second most abundant sequences identified for both fresh and dry manure samples. The 10 most abundant sequences comprised 18%, 35% and 28% of the total cloned sequences from aerosol, fresh and dry manure samples, respectively. Similar to the total cloned sequences, *Firmicutes* represented the highest percentage of the top 10 list (Table 2) of aerosol and fresh manure samples, whereas *Proteobacteria* topped the dry manure communities.

**Table 1:** Bacterial community dynamics of environmental samples collected from two dairies with distinct manure management practices.

Bacterial phylum	Percent total 16s rRNA sequences (no. of clones)							
	Sonoma dairy				Modesto dairy			
	Aerosol	Fresh manure	Dry manure	All	Aerosol	Fresh manure	Dry manure	All
*Firmicutes*	**55 (83)** D^a^	**52 (86)**	16 (28)	40 (197)	11 (12)	**57 (102)** A	33 (56) A	37 (170)
*Proteobacteria*	21 (31) F	1 (1)	**50 (90)** A	25 (122)	**78 (85)** FD	2 (4)	21 (37)	27 (126)
*Bacteroidetes*	14 (21)	42 (69) A	24 (43)	27 (133)	7 (8)	36 (65) A	4 (6)	17 (79)
*Actinobacteri*a	8 (12) F	0 (0)	9 (16)	6 (28)	1 (1)	1 (2)	27 (46) A	11 (49)
*Chloroflexi*	0 (0)	0 (0)	0 (0)	0 (0)	2 (2)	1 (1)	11 (19)	5 (22)
*Gemmatimonadetes*	0 (0)	0 (0)	0 (0)	0 (0)	0 (0)	0 (0)	5 (8)	2 (8)
*Spirochaetes*	1 (2)	5 (9)	0 (0)	2 (11)	0 (0)	3 (5)	0 (0)	1 (5)
*Tenericutes*	1 (1)	0 (0)	1 (2)	1 (3)	0 (0)	0 (0)	0 (0)	0 (0)
*Verrucomicrobia*	0 (0)	0 (0)	1 (1)	0 (1)	1 (1)	1 (1)	0 (0)	0 (2)

a Values shown in boldface type are ≥50% to show major differences in phyla represented between dairies and samples. Letter designations indicate that the numbers for these phyla are significantly different (*P*<0.01; Library compare) in samples of aerosols (A), fresh manure (F) or dry manure (D) collected from the same dairy.

**Table 2:** Classifier assignment of 10 abundant sequences from aerosol and manure samples from both dairies.

Bacterial phylum	Percent of top 10 abundant 16S rRNA sequences (no. of clones)					
	Sonoma dairy			Modesto dairy		
	Aerosol	Fresh manure	Dry manure	Aerosol	Fresh manure	Dry manure
*Firmicutes*	48 (13)	**60 (35)** a	0 (0)	0 (0)	**57 (32)**	0 (0)
*Bacteroidetes*	15 (4)	28 (16)	27 (14)	0 (0)	43 (24)	0 (0)
*Proteobacteria*	22 (6)	0 (0)	**73 (37)**	**100 (49)**	0 (0)	33 (18)
*Actinobacteria*	15 (4)	0 (0)	0 (0)	0 (0)	0 (0)	33 (18)
*Spirochaetes*	0 (0)	12 (7)	0 (0)	0 (0)	0 (0)	0 (0)
*Chloroflexi*	0 (0)	0 (0)	0 (0)	0 (0)	0 (0)	34 (19)

a Values shown in boldface type are ≥50% to show major differences in phyla represented between dairies and samples.

**Figure 2:**
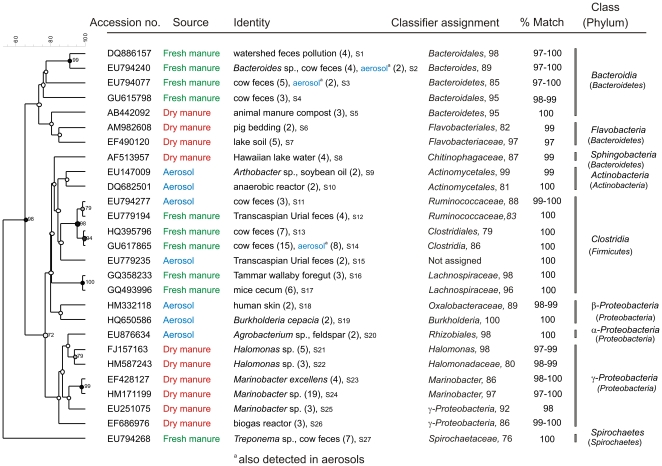
Phylogenetic relationship of 10 abundant 16S rRNA sequences from aerosol and manure samples from the Sonoma dairy. Identity or the original source of uncultured bacteria and the accession numbers are given. Numbers in parenthesis following the previous isolation source are number of clones characterized for the sequence. Numbers following the clones are identifiers for submissions to GenBank and sequence numbers S1 to S30 correspond with GenBank accession numbers HQ340004 to HQ340033. Bootstrap values >70% are noted at each node. Numbers following the Classifier assignment to the hierarchial taxa are percent reliability values for the classification.

Phylogenetic analysis of the 10 most abundant sequences indicates that members of *Clostridia* followed by *Bacteroidia* predominated in fresh manure (Figure 2). Identical sequences of members of *Bacteroidia* and *Clostridia* that were present in fresh manure were detected also from aerosols. These *Clostridia* had >94% sequence similarity with *Ruminococcus* sp. (S11 to S12; Figure 2) and *Clostridium* sp. (S13 and S14). However, all other sequences were unique to the source and were not found in other samples; 59% of all the top sequences were of fecal origin (Figure 2). Sequences of *Treponema* sp. previously characterized from cow feces constituted 12% of the top 10 sequences from fresh manure. Members of *Marinobacter* and *Halomonas* isolated predominantly from marine and saline habitats were exclusively detected from dry manure but not in fresh manure or aerosols (*P*<0.001).

### Bacterial communities in aerosol and manure samples from Modesto dairy

A total of 471 clones representing 7 different bacterial phyla were characterized from aerosol, fresh and dry manure samples collected from the Modesto dairy (Table 1). *Firmicutes* predominated both fresh and dry manure samples, whereas *Proteobacteria* predominated the bacterial communities in aerosols representing 78% of all sequences from aerosols. Members of *Bacteriodetes* and *Actinobacteria* were the second most abundant sequences identified from dry and fresh manure samples, respectively. *Chloroflexi* and *Gammatimonadetes* represented 11% and 5% of all sequences from dry manure.

**Figure 3:**
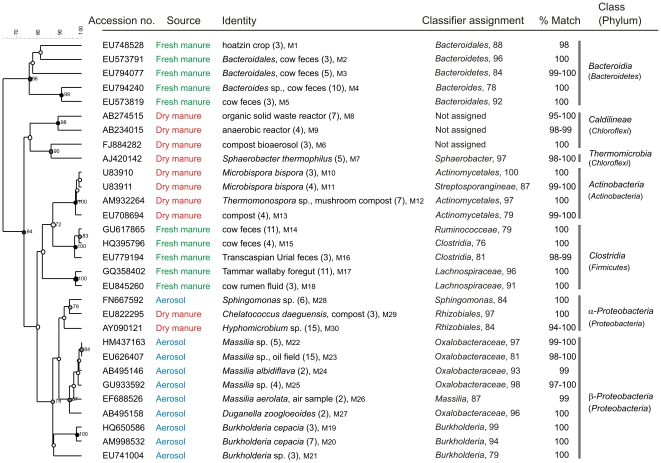
Phylogenetic relationship of 10 abundant 16S rRNA sequences from aerosol and manure samples from the Modesto dairy. Identity or the original source of uncultured bacteria and the accession numbers are given. Numbers in parenthesis following the source are number of clones characterized for the sequence. Numbers following the clones are identifiers for submission to GenBank and sequence numbers M1 to M30 correspond with GenBank accession numbers HQ340034 to HQ340063. Bootstrap values >70% are noted at each node. Numbers following the Classifier assignment to the hierarchial taxa are percent reliability values for the classification.

*Chloroflexi*, *Actinobacteria* and *Proteobacteria* sequences were represented equally in the top 10 abundant sequences from dry manure, compared to *Firmicutes* and *Bacteriodetes* in fresh manure; *Proteobacteria* was the only phylum detected in aerosols (Table 2). Members of *β-Proteobacteria* constituted 88% of the top 10 sequences from aerosols (Figure 3) and many of the sequences identified previously were associated with pollutant degradation. The 10 most abundant sequences from aerosol or manure samples were unique to the source and none of the top 10 for manure was detected in aerosols collected on the dairy. At least a third of the 10 abundant sequences were of fecal origin. All *Firmicutes* from the top 10 sequences in fresh manure were *Clostridia* that were previously isolated from animal feces (Figure 3).

### Rarefaction, species diversity and community comparisons from both dairies

**Figure 4:**
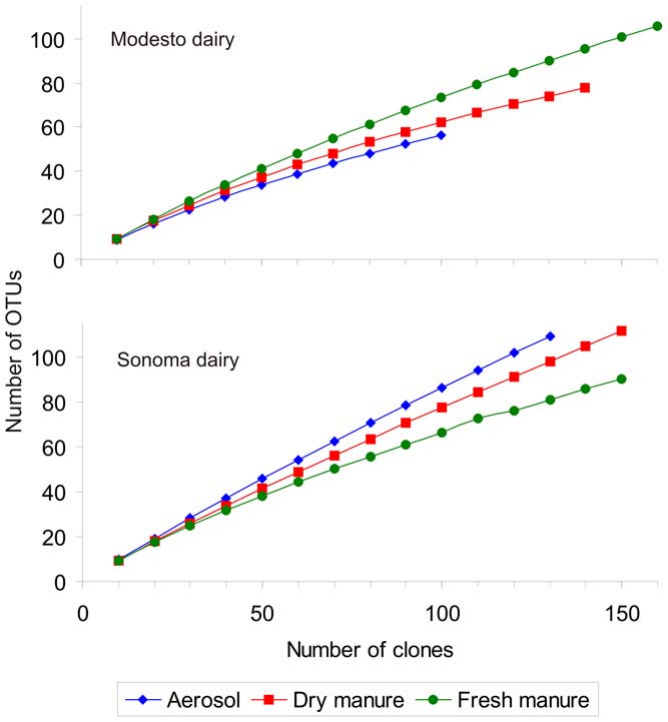
Rarefaction curves for 16S rRNA libraries of aerosol and manure samples from Modesto and Sonoma dairies. A total of 410 sequences representing 229 OTUs were used for rarefaction of clone libraries from Modesto (top panel), whereas 440 sequences representing 259 OTUs from Sonoma (bottom panel) were used.

Rarefaction estimates (Figure 4) using OTUs that shared >97% sequence similarity with GenBank accessions indicated that the clone libraries created from each environmental sample were not sufficient enough to fully explore the species diversity as indicated by the slope of the curves. However, species coverage estimates as high as 83.9% (Table 3) were obtained for bacterial communities in aerosols from Sonoma. Species diversity estimates indicate that aerosol communities from Sonoma were rich and even as compared to aerosol communities from the Modesto dairy (Table 3). Both Shannon-Wiener and Simpson's diversity index calculations yielded similar results. Drying the manure appeared to have reduced the species diversity at the Sonoma dairy and aerosolization further reduced the species diversity at the Modesto dairy.

**Table 3:** Species diversity and richness of bacterial communities in aerosol and manure samples from two different dairies.

Bacterial community source	Sonoma dairy^a^			Modesto dairy^a^		
	Shannon-Wiener	Simpson	Species coverage	Shannon-Wiener	Simpson	Species coverage
Aerosol	3.21	0.968	83.9%	3.01	0.937	56.4%
Fresh manure	3.14	0.952	60.1%	3.17	0.956	66.0%
Dry manure	2.97	0.916	74.4%	3.25	0.964	55.5%

a OTUs with >97% sequence similarity with GenBank accessions were used in determining the diversity indices and for rarefaction analysis. Species coverage estimates are calculated from rarefaction data.

**Figure 5:**
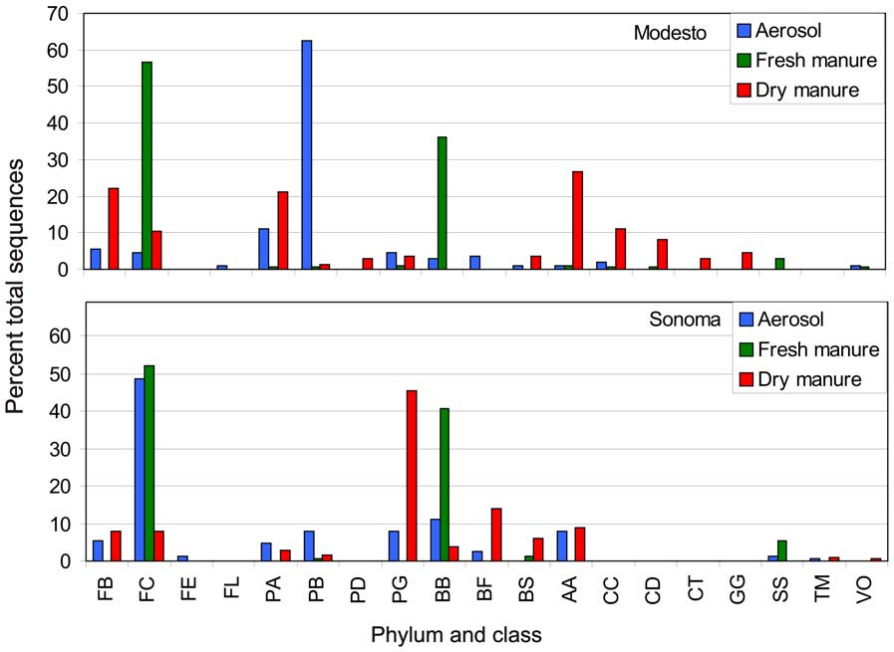
Percent distribution of all sequences from each isolation source assigned to bacterial classes. Top panel are sequences from Modesto dairy. Bottom panel are sequences from Sonoma dairy. Phylum class designations: FB  =  *Firmicutes-Bacilli*; FC  =  *Firmicutes-Clostridia*; FE  =  *Firmicutes-Erysipelotrichi*; PA  =  *α-Proteobacteria*; PB  =  *β-Proteobacteria*; PD  =  *δ-Proteobacteria*; PG  =  *γ-Proteobacteria*; BB  =  *Bacteroidetes-Bacteroidia*; BF  =  *Bacteroidetes-Flavobacteria*; BS  =  *Bacteroidetes-Sphingobacteria*; AA  =  *Actinobacteria-Actinobacteria*; CC  =  *Chloroflexi- Caldilineae*; CD  =  *Chloroflexi-Dehalococcoidetes*; CT  =  *Chloroflexi-Thermomicrobia*; GG  =  *Gemmatimonadetes-Gemmatimonadetes*; SS  =  *Spirochaetes-Spirochaetes*; TM  =  *Tenericutes-Mollicutes*; and VO  =  *Verrucomicrobia-Opitutae*.

Sequences that belong to 19 different bacterial classes were present in manure and aerosol samples from both dairies (Figure 5). Comparison of 16S rRNA sequences at OTU level using Library Compare indicate that members of class *β-Proteobacteria* that were predominant in aerosols (*P*<0.001) from Modesto did not originate from either fresh or dry manure. On the contrary, *Clostridia* were abundant in aerosols from Sonoma and members of *Lachnospiraceae* and *Ruminococcaceae* could have originated from fresh manure but none derived from dry manure (*P*<0.001). Members of *Clostridia* followed by *Bacteroidetes* were predominant in fresh manure from both dairies, but none of the members of class *Bacteroidia* in aerosols were from fresh manure (*P*<0.001). *Actinobacteria* followed by *Bacilli* dominated the bacteria in dry manure from the Modesto dairy, whereas *γ-Proteobacteria* and *Flavobacteria* were favored in dry manure from the Sonoma dairy (Figure 5). Members of class *Actinobacteria* characterized from aerosols of Modesto could have been derived from dry manure (*P* = 0.8) but not from fresh manure. Again, these data indicate the differences in the bacteria detected at different sites and manure compared to detectable bacteria in aerosols.

### Comparison of aerosol bacterial communities from spatially separated dairies

**Figure 6:**
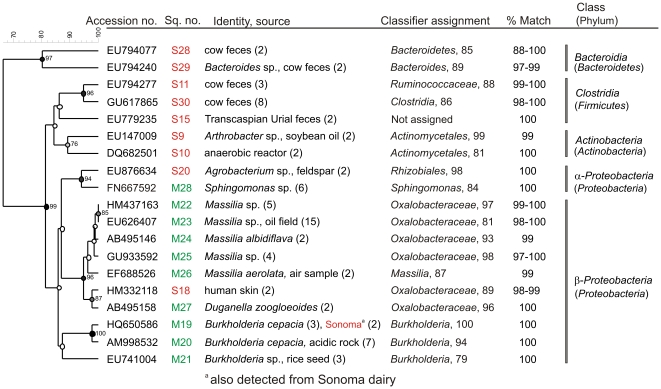
Phylogenetic relationship of the 10 abundant sequences characterized from aerosol samples collected from two different dairies. Identity or the original source of uncultured bacteria and the accession numbers are given. Sequence numbers (Sq no.) are GenBank identifiers for sequences from Sonoma dairy (S) and Modesto dairy (M). Numbers in parenthesis following the source are number of clones characterized for the sequence. Bootstrap values >70% are noted at each node. Classifier assignment to the hierarchial taxa along with percent reliability values are given.

Although the dairies are located 190 km from each other, we compared the aerosol bacterial communities for any similarities. The sequence similarities with GenBank sequences are significant; some of the 10 predominant sequences were assigned to >94% to genus level (Figure 6) by the Classifier software. Except for *Burkholderia cepacia* that was present in aerosols collected from both dairies all others were unique to each dairy (Figure 6). Taxonomic diversity of aerosol communities from Modesto was very low as 100% of the top ten were *Proteobacteria* and 42% of them belonged to genus *Massilia*; members of this genus were isolated previously from humans, polluted soils or air samples, but none of them were detected in manure from either dairy (*P*<0.001). In addition, 45% of all aerosol sequences were represented by the top 10 sequences. In contrast, taxonomic diversity of aerosol communities from the Sonoma dairy was very high and the clones identified representing the top 10 sequences (Figure 6) were only 18% of all sequences. Members of *Bacteriodetes*, *Firmicutes* and *Actinobacteria* represented 78% of the 10 abundant sequences of aerosols from Sonoma (Table 2) and almost half them were *Clostridia* (Figure 6).

### Culturable aerobic bacteria in aerosols and climate conditions

Aerosol-wash water samples used for bacterial community analysis from both dairies contained the same level of aerobic bacteria; the counts were 130±80 CFU/L of air per hour. In addition, aerobic counts from the Sonoma dairy remained the same from air samples collected on four different occasions during the months of May to August 2006 and the counts were 140±66 CFU/L of air per hour. Average temperature during samplings was 25±3°C and increased from 21±4°C at the beginning of samplings to 29±3°C towards the end. Wind speeds at the time of samplings averaged 3.5±1.6 kph and on one occasion during August 2006 the wind speed peaked at 13 kph. Humidity at the time of samplings was 43±12%.

## Discussion

We detected significant differences among bacterial communities in aerosols collected on-site in close proximity to dairy animals from two different dairies in California. This was achieved by using aerosol collection devices similar to the ones used for automated indoor pathogen detection systems [13], [14]. Low levels of aerobic bacteria were enumerated from Petrifilms, so we characterized bacterial communities from 97,200 liters of air from the Sonoma dairy and 129,600 liters of air from the Modesto dairy. These volumes are small compared to 1.4×10^6^ liters of air collected in an urban environment during a 24 h period to characterize >1 µm particles for bacteria [15]. We also maximized for the collection of bacteria that were likely from dairy cows by placing the aerosol devices in close proximity to hundreds of dairy cows in the barns. Since meteorological conditions are known to strongly influence the community structure of aerosols [16], aerosol wash-water from four devices was pooled to minimize the influence of wind, humidity and temperature fluctuations on the bacterial communities detected. Furthermore, location effects were also averaged by placing the collection devices in multiple locations on the dairy (Figure 1), and pooling the samples from all locations.

The detection of *Proteobacteria* representing 78% of all clones and 100% of the 10 most abundant sequences in aerosols from the Modesto dairy is noteworthy, because this phylum was nearly non-detectable in samples of fresh manure. A high proportion of *Proteobacteria* (81%) was detected also from aerosols collected downwind from a field treatment of biosolids [17]. The increase in proportion of *Proteobacteria* in aerosols (Table 2) is an indication of their non-fecal nature [18]. Drying in manure piles appears to have eliminated the most abundant sequences of phylum *Firmicutes* in fresh manure from both dairies (Table 2). Similarly, a high percentage of *Firmicutes* (75%) identified from dairy cow waste collected within 12 h after excretion [11] decreased to 10% in aerobic effluents of slurries prepared from the same waste. In contrast, *Firmicutes* were more abundant in aerosols and fresh manure from the Sonoma dairy compared to the Modesto dairy. *Firmicutes* were reported to be predominant also in aerosols produced during thermophilic composting [19]. Thus, these results indicate a dynamic nature of bacterial communities in aerosols from different dairies and are influenced by different environmental conditions.

The absence of sequences in aerosols originating from fresh or dry manure from the Modesto dairy (Figure 3) is noteworthy, especially considering our collection of aerosols in close proximity to cows on a dairy producing ∼35,000 kg of manure per day [2], plus the aerosols emanating presumably from the surrounding dairies. This result is consistent with data reported by Durso *et al* [20] that bacterial communities in a feed lot pen surface are separate and distinct from the feces collected from cattle in the same pen. However, 3 (uncharacterized *Bacteroidia* and *Clostridia*) out of the top 10 sequences characterized from the Sonoma dairy (Figure 2) originated from fresh manure and members of both phylogenetic classes have been proposed as surrogates for tracking fecal contamination [21], [22]. We attribute this result to the fine dust created by intense cattle movement around the aerosol collectors and to a more favorable wind direction (SSE 139° to 148°, SSW200°) at the Sonoma dairy, in contrast to a lack of apparent dust in the air nearer to the collectors at the Modesto dairy where the wind direction was NNE 13° to 26°.

Foodborne pathogens of high priority, such as *Salmonella*, *Campylobacter* and *Listeria*, were not detected from any of the 966 cloned sequences from aerosol and manure samples from both dairies. We detected one clone matching an *E. coli* in aerosols from the Sonoma dairy, but this is consistent with reported high concentrations of *E. coli* present in environmental samples on ranches and dairies [23]. Many of the unidentified sequences characterized from both dairies had been isolated previously from fecal sources (Figures 2 and 3). Gram-positive anaerobic *Clostridia*, some species of which are human pathogens, were present at a significant level (32% of top 10 sequences) in aerosols generated on the Sonoma dairy (Figures 5 and 6). These results are consistent with reports of *Clostridia* being predominant in aerosols from swine confinement facilities [24] and colonic contents of dairy cows [25]. Sequences representing the genus *Massilia* [26], and a possible human pathogen, were detected in aerosols from both dairies. *Burkholderia cepacia*, found in aerosols from both dairies is a human pathogen linked with outbreaks particularly among cystic fibrosis patients and has been developed as a biopesticide and for bioremediation [27]. It was detected also in a previous study of urban aerosols collected from a site in Albany, California [28]. *Duganella zoogloeoides*, a possible biocontrol agent, was detected from the Modesto dairy aerosol communities. Fresh manure from the Sonoma dairy contained possible animal or human pathogens of genus *Treponema* that were also detected from fecal microbiota obtained from a cattle herd with endemic *Salmonella* [29]. Dry manure from Modesto contained a sequence that matched 100% with the GenBank sequence of potentially pathogenic slow growing *Mycobacterium* sp. [30] and >96% sequence similarity with *Mycobacterium avium paratuberculosis* that causes debilitating Johne's disease in cattle and known to be associated with human Crohn's disease [31]. This is of particular importance since 27% of library sequences from Modesto dry manure and 8% of sequences in aerosols from Sonoma belong to *Actinobacteria* and, thus, a potential exists for the aerosol transport of *Mycobacterium avium paratuberculosis*, which has been reported to survive in feces for more than a year [32], and has been isolated from two-thirds of manure lagoons [33]. Many other potential human and animal pathogens were detected at a single clone level from aerosols and manure samples from both dairies, but the concentration and potential viability of these organisms is uncertain.

In summary, we are reporting the first study of bacterial communities in aerosols from dairies in California. The bacteria in aerosols from the Modesto dairy did not originate from either fresh or dry manure samples collected on-site, a result in contrast to at least three abundant sequences detected in Sonoma dairy aerosols originating from fresh manure. Since, most bacterial sequences detected from aerosols or manure were unique to the sample source, further studies of aerosols from more dairies in the same or different locations will be required to determine the variability in bacterial communities in aerosols, whether bacteria in aerosols have enhanced fitness for survival in that environment, and whether any sequences can be identified as surrogates for tracking transport of enteric pathogens from point sources (e.g., ranches/dairies) to raw produce crops grown in the same vicinity.

## Supporting Information

Figure S1 Comparison of OTU libraries from aerosols with libraries generated from fresh and dry manure samples collected from Sonoma and Modesto dairies using RDP Library Compare. OTU libraries generated from aerosols from both dairies were also compared.
(PDF)
