# Supplementary material for: Bacterial Communities in Aerosols and Manure Samples from Two Different Dairies in Central and Sonoma Valleys of California
Source: PLoS One. 2011 Feb 18;6(2):e17281. doi: 10.1371/journal.pone.0017281 (PMC3041799; doi:10.1371/journal.pone.0017281)
Supplement: Figure S1 — Comparison of OTU libraries from aerosols with libraries generated from fresh and dry manure samples collected from Sonoma and Modesto dairies using RDP Library Compare. OTU libraries generated from aerosols from both dairies were also compared. (PDF) [file pone.0017281.s001.pdf]

Library Comparison Summary

[start over | tabular view | help]

Library Compare: RDP Naive Bayesian rRNA Classifier Version 2.2, March 2010
Taxonomical Hierarchy: RDP training set 6, based on nomenclatural taxonomy and Bergey's Manual
Query Submit Date: Fri Dec 17 13:49:10 EST 2010

Library1: *AM8 son aerosols.txt* Total # of sequences: 147
Library2: *AM8 son fresh.txt* Total # of sequences: 169

Display depth: 10 **Confidence threshold:** 80% Refresh

Library1	%	phylum	%	Library2
	1.4	"Spirochaetes"	0.6	
	6.8	"Actinobacteria" *	0.0	
	13.6	"Bacteroidetes" *	39.1	
	34.7	"Firmicutes"	33.7	
	19.7	"Proteobacteria" *	1.2	
	23.8	unclassified Bacteria	25.4	

(* = significantly different at 0.01)

Lineage *(click to return to particular node)*:

Root (147/169/9.96E-1)

Hierarchy View *(click a node to make it the root -- click the root to see sequence assignment detail)*:

domain Bacteria (147/169/9.96E-1) (library1/library2/significance value) [*show assignment detail*]
» » phylum "Spirochaetes" (2/1/5.24E-1)
» » » class Spirochaetes (2/1/5.24E-1)
» » » » order Spirochaetales (2/1/5.24E-1)
» » » » » family Spirochaetaceae (2/1/5.24E-1)
» » » » » » genus Treponema (1/1/8.96E-1)
» » » » » » unclassified Spirochaetaceae (1/0/NA)
» » phylum "Actinobacteria" (10/0/4.42E-4)
» » » class Actinobacteria (10/0/4.42E-4)
» » » » subclass Actinobacteridae (9/0/9.49E-4)
» » » » » order Actinomycetales (9/0/9.49E-4)
» » » » » » suborder Propionibacterineae (1/0/4.33E-1)
» » » » » » » family Nocardioidaceae (1/0/4.33E-1)
» » » » » » » » genus Nocardioides (1/0/4.33E-1)
» » » » » » suborder Micrococcineae (2/0/2.01E-1)
» » » » » » » unclassified Micrococcineae (2/0/NA)
» » » » » » suborder Corynebacterineae (2/0/2.01E-1)
» » » » » » » family Corynebacteriaceae (1/0/4.33E-1)
» » » » » » » » genus Corynebacterium (1/0/4.33E-1)
» » » » » » » unclassified Corynebacterineae (1/0/NA)
» » » » » » unclassified Actinomycetales (4/0/NA)
» » » » unclassified Actinobacteria (1/0/NA)
» » phylum "Bacteroidetes" (20/66/5.74E-7)
» » » class "Sphingobacteria" (0/2/3.06E-1)
» » » » order "Sphingobacteriales" (0/2/3.06E-1)
» » » » » unclassified "Sphingobacteriales" (0/2/NA)
» » » class Flavobacteria (4/0/4.36E-2)
» » » » order "Flavobacteriales" (4/0/4.36E-2)
» » » » » family Flavobacteriaceae (2/0/2.01E-1)
» » » » » » unclassified Flavobacteriaceae (2/0/NA)
» » » » » unclassified "Flavobacteriales" (2/0/NA)
» » » class "Bacteroidia" (7/30/3.4E-4)
» » » » order "Bacteroidales" (7/30/3.4E-4)
» » » » » family "Rikenellaceae" (0/2/3.06E-1)
» » » » » » genus Alistipes (0/1/5.72E-1)
» » » » » » unclassified "Rikenellaceae" (0/1/NA)
» » » » » family "Prevotellaceae" (0/1/5.72E-1)
» » » » » » genus Prevotella (0/1/5.72E-1)
» » » » » family Bacteroidaceae (0/6/2.5E-2)
» » » » » » genus Bacteroides (0/6/2.5E-2)
» » » » » family "Porphyromonadaceae" (0/1/5.72E-1)
» » » » » » unclassified "Porphyromonadaceae" (0/1/NA)
» » » » » unclassified "Bacteroidales" (7/20/NA)
» » » unclassified "Bacteroidetes" (9/34/NA)
» » phylum "Firmicutes" (51/57/8.57E-1)
» » » class "Bacilli" (7/0/4.39E-3)
» » » » order Bacillales (6/0/9.43E-3)
» » » » » family Caryophanaceae (1/0/4.33E-1)
» » » » » » genus Caryophanon (1/0/4.33E-1)
» » » » » family "Staphylococcaceae" (3/0/9.37E-2)
» » » » » » genus Staphylococcus (1/0/4.33E-1)
» » » » » » genus Jeotgalicoccus (2/0/2.01E-1)
» » » » » unclassified Bacillales (2/0/NA)
» » » » unclassified "Bacilli" (1/0/NA)
» » » class "Erysipelotrichi" (2/0/2.01E-1)
» » » » order "Erysipelotrichales" (2/0/2.01E-1)
» » » » » family Erysipelotrichaceae (2/0/2.01E-1)
» » » » » » genus Turicibacter (2/0/2.01E-1)
» » » class "Clostridia" (42/56/3.79E-1)
» » » » order Clostridiales (36/45/6.67E-1)
» » » » » family Veillonellaceae (1/0/4.33E-1)
» » » » » » unclassified Veillonellaceae (1/0/NA)
» » » » » family Clostridiaceae (1/0/4.33E-1)
» » » » » » subfamily "Clostridiaceae 1" (1/0/4.33E-1)
» » » » » » » unclassified "Clostridiaceae 1" (1/0/NA)
» » » » » family "Lachnospiraceae" (13/25/1.05E-1)
» » » » » » genus Butyrivibrio (0/1/5.72E-1)
» » » » » » unclassified "Lachnospiraceae" (13/24/NA)
» » » » » family "Ruminococcaceae" (7/5/4.19E-1)
» » » » » » genus Oscillibacter (1/0/4.33E-1)
» » » » » » genus Papillibacter (1/0/4.33E-1)
» » » » » » unclassified "Ruminococcaceae" (5/5/NA)
» » » » » unclassified Clostridiales (14/15/NA)
» » » » unclassified "Clostridia" (6/11/NA)
» » » unclassified "Firmicutes" (0/1/NA)
» » phylum "Proteobacteria" (29/2/3.21E-8)
» » » class Alphaproteobacteria (7/0/4.39E-3)
» » » » order Rhizobiales (5/0/2.03E-2)
» » » » » unclassified Rhizobiales (5/0/NA)
» » » » order Rhodospirillales (1/0/4.33E-1)
» » » » » family Rhodospirillaceae (1/0/4.33E-1)
» » » » » » unclassified Rhodospirillaceae (1/0/NA)
» » » » order Sphingomonadales (1/0/4.33E-1)
» » » » » family Sphingomonadaceae (1/0/4.33E-1)
» » » » » » genus Sphingomonas (1/0/4.33E-1)
» » » class Betaproteobacteria (10/1/3.04E-3)
» » » » order Burkholderiales (10/1/3.04E-3)
» » » » » family Alcaligenaceae (1/1/8.96E-1)
» » » » » » genus Sutterella (1/1/8.96E-1)
» » » » » family Oxalobacteraceae (3/0/9.37E-2)
» » » » » » unclassified Oxalobacteraceae (3/0/NA)
» » » » » family Burkholderiaceae (5/0/2.03E-2)
» » » » » » genus Burkholderia (4/0/4.36E-2)
» » » » » » unclassified Burkholderiaceae (1/0/NA)
» » » » » family Comamonadaceae (1/0/4.33E-1)
» » » » » » unclassified Comamonadaceae (1/0/NA)
» » » class Gammaproteobacteria (11/0/2.05E-4)
» » » » order Xanthomonadales (2/0/2.01E-1)
» » » » » family Xanthomonadaceae (2/0/2.01E-1)
» » » » » » genus Luteimonas (1/0/4.33E-1)
» » » » » » genus Frateuria (1/0/4.33E-1)
» » » » order "Enterobacteriales" (1/0/4.33E-1)
» » » » » family Enterobacteriaceae (1/0/4.33E-1)
» » » » » » genus Escherichia/Shigella (1/0/4.33E-1)
» » » » order Pseudomonadales (2/0/2.01E-1)
» » » » » family Pseudomonadaceae (2/0/2.01E-1)
» » » » » » genus Pseudomonas (1/0/4.33E-1)
» » » » » » unclassified Pseudomonadaceae (1/0/NA)
» » » » unclassified Gammaproteobacteria (6/0/NA)
» » » unclassified "Proteobacteria" (1/1/NA)
» » unclassified Bacteria (35/43/NA)

© 1992-2009 Michigan State University Board of Trustees, East Lansing MI 48824
MSU is an affirmative-action, equal-opportunity employer.

Library Comparison Summary

[start over | tabular view | help]

Library Compare: RDP Naive Bayesian rRNA Classifier Version 2.2, March 2010
Taxonomical Hierarchy: RDP training set 6, based on nomenclatural taxonomy and Bergey's Manual
Query Submit Date: Fri Dec 17 13:47:23 EST 2010

Library1: *AM8 son aerosols.txt* Total # of sequences: 147
Library2: *AM8 son dry.txt* Total # of sequences: 185

Display depth: 10 **Confidence threshold:** 80% Refresh

Library1	%	phylum	%	Library2
	0.0	"Verrucomicrobia"	0.5	
	0.0	TM7	0.5	
	0.0	"Tenericutes"	1.1	
	0.7	"Spirochaetes"	0.0	
	6.8	"Actinobacteria"	7.6	
	13.6	"Bacteroidetes"	20.5	
	33.3	"Firmicutes" *	10.8	
	19.7	"Proteobacteria" *	47.6	
	25.9	unclassified Bacteria	11.4	

(* = significantly different at 0.01)

Lineage ***(click to return to particular node):***

Root (147/185/9.93E-1)

Hierarchy View ***(click a node to make it the root -- click the root to see sequence assignment detail):***

domain Bacteria (147/185/9.93E-1) (library1/library2/significance value) [*show assignment detail*]
» » phylum "Verrucomicrobia" (0/1/6.21E-1)
» » » class Opitutae (0/1/6.21E-1)
» » » » unclassified Opitutae (0/1/NA)
» » phylum TM7 (0/1/6.21E-1)
» » » genus TM7_genera_incertae_sedis (0/1/6.21E-1)
» » phylum "Tenericutes" (0/2/3.46E-1)
» » » class Mollicutes (0/2/3.46E-1)
» » » » order Acholeplasmatales (0/2/3.46E-1)
» » » » » family Acholeplasmataceae (0/2/3.46E-1)
» » » » » » genus Acholeplasma (0/2/3.46E-1)
» » phylum "Spirochaetes" (1/0/3.92E-1)
» » » class Spirochaetes (1/0/3.92E-1)
» » » » order Spirochaetales (1/0/3.92E-1)
» » » » » family Spirochaetaceae (1/0/3.92E-1)
» » » » » » genus Treponema (1/0/3.92E-1)
» » phylum "Actinobacteria" (10/14/7.87E-1)
» » » class Actinobacteria (10/14/7.87E-1)
» » » » subclass Rubrobacteridae (0/1/6.21E-1)
» » » » » unclassified Rubrobacteridae (0/1/NA)
» » » » subclass Actinobacteridae (9/10/7.79E-1)
» » » » » order Actinomycetales (9/10/7.79E-1)
» » » » » » suborder Streptosporangineae (0/1/6.21E-1)
» » » » » » » family Nocardiopsaceae (0/1/6.21E-1)
» » » » » » » » genus Thermobifida (0/1/6.21E-1)
» » » » » » suborder Propionibacterineae (1/0/3.92E-1)
» » » » » » » family Nocardioidaceae (1/0/3.92E-1)
» » » » » » » » genus Nocardioides (1/0/3.92E-1)
» » » » » » suborder Micrococcineae (1/2/8.06E-1)
» » » » » » » family Microbacteriaceae (0/1/6.21E-1)
» » » » » » » » unclassified Microbacteriaceae (0/1/NA)
» » » » » » » unclassified Micrococcineae (1/1/NA)
» » » » » » suborder Corynebacterineae (2/2/7.87E-1)
» » » » » » » family Corynebacteriaceae (1/2/8.06E-1)
» » » » » » » » genus Corynebacterium (1/2/8.06E-1)
» » » » » » » unclassified Corynebacterineae (1/0/NA)
» » » » » » unclassified Actinomycetales (5/5/NA)
» » » » unclassified Actinobacteria (1/3/NA)
» » phylum "Bacteroidetes" (20/38/9.89E-2)
» » » class "Sphingobacteria" (0/6/3.34E-2)
» » » » order "Sphingobacteriales" (0/6/3.34E-2)
» » » » » family Sphingobacteriaceae (0/1/6.21E-1)
» » » » » » unclassified Sphingobacteriaceae (0/1/NA)
» » » » » family "Chitinophagaceae" (0/5/5.99E-2)
» » » » » » genus Gracilimonas (0/1/6.21E-1)
» » » » » » unclassified "Chitinophagaceae" (0/4/NA)
» » » class Flavobacteria (4/14/6.44E-2)
» » » » order "Flavobacteriales" (4/14/6.44E-2)
» » » » » family Flavobacteriaceae (2/12/2.46E-2)
» » » » » » unclassified Flavobacteriaceae (2/12/NA)
» » » » » unclassified "Flavobacteriales" (2/2/NA)
» » » class "Bacteroidia" (7/3/1.1E-1)
» » » » order "Bacteroidales" (7/3/1.1E-1)
» » » » » family "Porphyromonadaceae" (1/3/5.34E-1)
» » » » » » genus Proteiniphilum (0/1/6.21E-1)
» » » » » » unclassified "Porphyromonadaceae" (1/2/NA)
» » » » » unclassified "Bacteroidales" (6/0/NA)
» » » unclassified "Bacteroidetes" (9/15/NA)
» » phylum "Firmicutes" (49/20/5.74E-7)
» » » class "Bacilli" (7/12/5.03E-1)
» » » » order "Lactobacillales" (0/1/6.21E-1)
» » » » » family "Carnobacteriaceae" (0/1/6.21E-1)
» » » » » » genus Atopostipes (0/1/6.21E-1)
» » » » order Bacillales (6/9/7.34E-1)
» » » » » family Planococcaceae (0/1/6.21E-1)
» » » » » » genus Planococcus (0/1/6.21E-1)
» » » » » family Bacillaceae (0/3/1.93E-1)
» » » » » » unclassified Bacillaceae (0/3/NA)
» » » » » family Caryophanaceae (1/0/3.92E-1)
» » » » » » genus Caryophanon (1/0/3.92E-1)
» » » » » family "Staphylococcaceae" (3/2/4.87E-1)
» » » » » » genus Salinicoccus (0/1/6.21E-1)
» » » » » » genus Staphylococcus (1/0/3.92E-1)
» » » » » » genus Jeotgalicoccus (2/1/4.64E-1)
» » » » » unclassified Bacillales (2/3/NA)
» » » » unclassified "Bacilli" (1/2/NA)
» » » class "Erysipelotrichi" (2/0/1.74E-1)
» » » » order "Erysipelotrichales" (2/0/1.74E-1)
» » » » » family Erysipelotrichaceae (2/0/1.74E-1)
» » » » » » genus Turicibacter (2/0/1.74E-1)
» » » class "Clostridia" (39/7/3.65E-9)
» » » » order Clostridiales (32/6/2E-7)
» » » » » family Veillonellaceae (2/0/1.74E-1)
» » » » » » unclassified Veillonellaceae (2/0/NA)
» » » » » family Clostridiaceae (1/0/3.92E-1)
» » » » » » subfamily "Clostridiaceae 1" (1/0/3.92E-1)
» » » » » » » unclassified "Clostridiaceae 1" (1/0/NA)
» » » » » family "Lachnospiraceae" (13/0/2.23E-5)
» » » » » » genus Butyrivibrio (1/0/3.92E-1)
» » » » » » unclassified "Lachnospiraceae" (12/0/NA)
» » » » » family "Ruminococcaceae" (5/0/1.51E-2)
» » » » » » genus Oscillibacter (1/0/3.92E-1)
» » » » » » unclassified "Ruminococcaceae" (4/0/NA)
» » » » » unclassified Clostridiales (11/6/NA)
» » » » unclassified "Clostridia" (7/1/NA)
» » » unclassified "Firmicutes" (1/1/NA)
» » phylum "Proteobacteria" (29/88/2E-7)
» » » class Alphaproteobacteria (7/4/2.04E-1)
» » » » order Rhodobacterales (0/1/6.21E-1)
» » » » » family Rhodobacteraceae (0/1/6.21E-1)
» » » » » » unclassified Rhodobacteraceae (0/1/NA)
» » » » order Rhizobiales (5/1/6.55E-2)
» » » » » family Methylobacteriaceae (1/0/3.92E-1)
» » » » » » genus Methylobacterium (1/0/3.92E-1)
» » » » » unclassified Rhizobiales (4/1/NA)
» » » » order Rhodospirillales (1/0/3.92E-1)
» » » » » family Rhodospirillaceae (1/0/3.92E-1)
» » » » » » genus Azospirillum (1/0/3.92E-1)
» » » » order Sphingomonadales (1/0/3.92E-1)
» » » » » family Sphingomonadaceae (1/0/3.92E-1)
» » » » » » genus Sphingomonas (1/0/3.92E-1)
» » » » unclassified Alphaproteobacteria (0/2/NA)
» » » class Betaproteobacteria (10/2/7.08E-3)
» » » » order Burkholderiales (10/1/1.83E-3)
» » » » » family Alcaligenaceae (1/1/8.29E-1)
» » » » » » genus Sutterella (1/0/3.92E-1)
» » » » » » unclassified Alcaligenaceae (0/1/NA)
» » » » » family Oxalobacteraceae (3/0/7.69E-2)
» » » » » » unclassified Oxalobacteraceae (3/0/NA)
» » » » » family Burkholderiaceae (5/0/1.51E-2)
» » » » » » genus Burkholderia (3/0/7.69E-2)
» » » » » » unclassified Burkholderiaceae (2/0/NA)
» » » » » family Comamonadaceae (1/0/3.92E-1)
» » » » » » genus Variovorax (1/0/3.92E-1)
» » » » unclassified Betaproteobacteria (0/1/NA)
» » » class Gammaproteobacteria (11/69/2.99E-10)
» » » » order Aeromonadales (0/1/6.21E-1)
» » » » » family Aeromonadaceae (0/1/6.21E-1)
» » » » » » genus Oceanimonas (0/1/6.21E-1)
» » » » order Alteromonadales (0/16/9.63E-5)
» » » » » family Alteromonadaceae (0/14/3.1E-4)
» » » » » » genus Marinobacter (0/10/3.22E-3)
» » » » » » unclassified Alteromonadaceae (0/4/NA)
» » » » » unclassified Alteromonadales (0/2/NA)
» » » » order Xanthomonadales (2/0/1.74E-1)
» » » » » family Xanthomonadaceae (2/0/1.74E-1)
» » » » » » genus Luteimonas (1/0/3.92E-1)
» » » » » » genus Frateuria (1/0/3.92E-1)
» » » » order "Enterobacteriales" (2/0/1.74E-1)
» » » » » family Enterobacteriaceae (2/0/1.74E-1)
» » » » » » genus Escherichia/Shigella (1/0/3.92E-1)
» » » » » » unclassified Enterobacteriaceae (1/0/NA)
» » » » order Pseudomonadales (2/1/4.64E-1)
» » » » » family Pseudomonadaceae (1/1/8.29E-1)
» » » » » » genus Pseudomonas (1/0/3.92E-1)
» » » » » » unclassified Pseudomonadaceae (0/1/NA)
» » » » » unclassified Pseudomonadales (1/0/NA)
» » » » order Oceanospirillales (0/9/5.77E-3)
» » » » » family Halomonadaceae (0/9/5.77E-3)
» » » » » » genus Halomonas (0/8/1.04E-2)
» » » » » » unclassified Halomonadaceae (0/1/NA)
» » » » unclassified Gammaproteobacteria (5/42/NA)
» » » unclassified "Proteobacteria" (1/13/NA)
» » unclassified Bacteria (38/21/NA)

© 1992-2009 Michigan State University Board of Trustees, East Lansing MI 48824
MSU is an affirmative-action, equal-opportunity employer.

Library Comparison Summary

[start over | tabular view | help]

Library Compare: RDP Naive Bayesian rRNA Classifier Version 2.2, March 2010
Taxonomical Hierarchy: RDP training set 6, based on nomenclatural taxonomy and Bergey's Manual
Query Submit Date: Fri Dec 17 13:42:56 EST 2010

Library1: *AM8 mod aerosols.txt* Total # of sequences: 108
Library2: *AM8 mod fresh.txt* Total # of sequences: 176

Display depth: 10 **Confidence threshold:** 80% Refresh

Library1	%	phylum	%	Library2
	0.0	"Spirochaetes"	1.7	
	0.9	"Actinobacteria"	1.1	
	0.9	"Verrucomicrobia"	0.0	
	6.5	"Firmicutes" *	33.5	
	0.9	"Acidobacteria"	0.0	
	7.4	"Bacteroidetes" *	35.8	
	75.9	"Proteobacteria" *	0.6	
	7.4	unclassified Bacteria	27.3	

(* = significantly different at 0.01)

Lineage *(click to return to particular node)*:

Root (108/176/9.84E-1)

Hierarchy View *(click a node to make it the root -- click the root to see sequence assignment detail)*:

domain Bacteria (108/176/9.84E-1) (library1/library2/significance value) [*show assignment detail*]
» » phylum "Spirochaetes" (0/3/2.95E-1)
» » » class Spirochaetes (0/3/2.95E-1)
» » » » order Spirochaetales (0/3/2.95E-1)
» » » » » family Spirochaetaceae (0/2/4.76E-1)
» » » » » » genus Treponema (0/2/4.76E-1)
» » » » » unclassified Spirochaetales (0/1/NA)
» » phylum "Actinobacteria" (1/2/1.02E0)
» » » class Actinobacteria (1/2/1.02E0)
» » » » subclass Actinobacteridae (1/2/1.02E0)
» » » » » order Bifidobacteriales (0/2/4.76E-1)
» » » » » » family Bifidobacteriaceae (0/2/4.76E-1)
» » » » » » » genus Bifidobacterium (0/1/7.68E-1)
» » » » » » » unclassified Bifidobacteriaceae (0/1/NA)
» » » » » order Actinomycetales (1/0/2.89E-1)
» » » » » » unclassified Actinomycetales (1/0/NA)
» » phylum "Verrucomicrobia" (1/0/2.89E-1)
» » » unclassified "Verrucomicrobia" (1/0/NA)
» » phylum "Firmicutes" (7/59/2E-7)
» » » class "Clostridia" (0/54/7.44E-12)
» » » » order Clostridiales (0/45/5.52E-10)
» » » » » family Clostridiaceae (0/1/7.68E-1)
» » » » » » subfamily "Clostridiaceae 1" (0/1/7.68E-1)
» » » » » » » unclassified "Clostridiaceae 1" (0/1/NA)
» » » » » family "Lachnospiraceae" (0/28/1.88E-6)
» » » » » » unclassified "Lachnospiraceae" (0/28/NA)
» » » » » family Veillonellaceae (0/1/7.68E-1)
» » » » » » unclassified Veillonellaceae (0/1/NA)
» » » » » family "Ruminococcaceae" (0/3/2.95E-1)
» » » » » » unclassified "Ruminococcaceae" (0/3/NA)
» » » » » unclassified Clostridiales (0/12/NA)
» » » » unclassified "Clostridia" (0/9/NA)
» » » class "Bacilli" (6/0/2.3E-3)
» » » » order "Lactobacillales" (1/0/2.89E-1)
» » » » » family "Carnobacteriaceae" (1/0/2.89E-1)
» » » » » » genus Atopostipes (1/0/2.89E-1)
» » » » order Bacillales (3/0/4.18E-2)
» » » » » family Caryophanaceae (1/0/2.89E-1)
» » » » » » genus Caryophanon (1/0/2.89E-1)
» » » » » family "Staphylococcaceae" (1/0/2.89E-1)
» » » » » » genus Salinicoccus (1/0/2.89E-1)
» » » » » family Bacillaceae (1/0/2.89E-1)
» » » » » » unclassified Bacillaceae (1/0/NA)
» » » » unclassified "Bacilli" (2/0/NA)
» » » unclassified "Firmicutes" (1/5/NA)
» » phylum "Acidobacteria" (1/0/2.89E-1)
» » » unclassified "Acidobacteria" (1/0/NA)
» » phylum "Bacteroidetes" (8/63/1.16E-7)
» » » class "Bacteroidia" (2/38/2.02E-6)
» » » » order "Bacteroidales" (2/38/2.02E-6)
» » » » » family "Prevotellaceae" (0/4/1.83E-1)
» » » » » » genus Prevotella (0/2/4.76E-1)
» » » » » » unclassified "Prevotellaceae" (0/2/NA)
» » » » » family "Porphyromonadaceae" (0/1/7.68E-1)
» » » » » » unclassified "Porphyromonadaceae" (0/1/NA)
» » » » » family "Rikenellaceae" (0/2/4.76E-1)
» » » » » » unclassified "Rikenellaceae" (0/2/NA)
» » » » » family Bacteroidaceae (0/8/2.7E-2)
» » » » » » genus Bacteroides (0/8/2.7E-2)
» » » » » unclassified "Bacteroidales" (2/23/NA)
» » » class Flavobacteria (1/1/6.48E-1)
» » » » order "Flavobacteriales" (1/1/6.48E-1)
» » » » » family Flavobacteriaceae (1/1/6.48E-1)
» » » » » » unclassified Flavobacteriaceae (1/1/NA)
» » » class "Sphingobacteria" (1/0/2.89E-1)
» » » » order "Sphingobacteriales" (1/0/2.89E-1)
» » » » » unclassified "Sphingobacteriales" (1/0/NA)
» » » unclassified "Bacteroidetes" (4/24/NA)
» » phylum "Proteobacteria" (82/1/1.48E-33)
» » » class Alphaproteobacteria (11/0/1.83E-5)
» » » » order Caulobacterales (1/0/2.89E-1)
» » » » » family Caulobacteraceae (1/0/2.89E-1)
» » » » » » unclassified Caulobacteraceae (1/0/NA)
» » » » order Rhizobiales (3/0/4.18E-2)
» » » » » family Methylobacteriaceae (2/0/1.1E-1)
» » » » » » genus Methylobacterium (2/0/1.1E-1)
» » » » » unclassified Rhizobiales (1/0/NA)
» » » » order Sphingomonadales (7/0/8.75E-4)
» » » » » family Sphingomonadaceae (7/0/8.75E-4)
» » » » » » genus Sphingomonas (6/0/2.3E-3)
» » » » » » unclassified Sphingomonadaceae (1/0/NA)
» » » class Gammaproteobacteria (4/1/6.52E-2)
» » » » order Chromatiales (1/0/2.89E-1)
» » » » » unclassified Chromatiales (1/0/NA)
» » » » order Pseudomonadales (2/0/1.1E-1)
» » » » » family Pseudomonadaceae (1/0/2.89E-1)
» » » » » » unclassified Pseudomonadaceae (1/0/NA)
» » » » » family Moraxellaceae (1/0/2.89E-1)
» » » » » » genus Psychrobacter (1/0/2.89E-1)
» » » » unclassified Gammaproteobacteria (1/1/NA)
» » » class Betaproteobacteria (64/0/1.02E-27)
» » » » order Burkholderiales (62/0/7.04E-27)
» » » » » family Comamonadaceae (8/0/3.33E-4)
» » » » » » genus Variovorax (7/0/8.75E-4)
» » » » » » unclassified Comamonadaceae (1/0/NA)
» » » » » family Oxalobacteraceae (37/0/2.21E-16)
» » » » » » genus Massilia (10/0/4.81E-5)
» » » » » » unclassified Oxalobacteraceae (27/0/NA)
» » » » » family Burkholderiaceae (15/0/3.83E-7)
» » » » » » genus Burkholderia (15/0/3.83E-7)
» » » » » unclassified Burkholderiales (2/0/NA)
» » » » unclassified Betaproteobacteria (2/0/NA)
» » » unclassified "Proteobacteria" (3/0/NA)
» » unclassified Bacteria (8/48/NA)

CENTER FOR MICROBIAL ECOLOGY @ Michigan State University

MICHIGAN STATE UNIVERSITY Advancing Knowledge. Transforming Lives.

© 1992-2009 Michigan State University Board of Trustees, East Lansing MI 48824
MSU is an affirmative-action, equal-opportunity employer.

Library Comparison Summary

[start over | tabular view | help]

Library Compare: RDP Naive Bayesian rRNA Classifier Version 2.2, March 2010
Taxonomical Hierarchy: RDP training set 6, based on nomenclatural taxonomy and Bergey's Manual
Query Submit Date: Fri Dec 17 12:18:48 EST 2010

Library1: *AM8 mod aerosols.txt* Total # of sequences: 108
Library2: *AM8 mod dry.txt* Total # of sequences: 167

Display depth: 10 **Confidence threshold:** 80% Refresh

Library1	%	phylum	%	Library2
	0.0	"Chloroflexi"	3.0	
	0.9	"Actinobacteria" *	21.0	
	0.9	"Verrucomicrobia"	0.0	
	6.5	"Firmicutes" *	18.0	
	0.9	"Acidobacteria"	0.6	
	7.4	"Bacteroidetes"	2.4	
	75.9	"Proteobacteria" *	15.0	
	7.4	unclassified Bacteria	40.1	

(* = significantly different at 0.01)

Lineage *(click to return to particular node)*:

Root (108/167/9.86E-1)

Hierarchy View *(click a node to make it the root -- click the root to see sequence assignment detail)*:

domain Bacteria (108/167/9.86E-1) (library1/library2/significance value) [*show assignment detail*]
» » phylum "Chloroflexi" (0/5/1E-1)
» » » class Thermomicrobia (0/5/1E-1)
» » » » subclass Sphaerobacteridae (0/5/1E-1)
» » » » » order Sphaerobacterales (0/5/1E-1)
» » » » » » suborder "Sphaerobacterineae" (0/5/1E-1)
» » » » » » » family Sphaerobacteraceae (0/5/1E-1)
» » » » » » » » genus Sphaerobacter (0/5/1E-1)
» » phylum "Actinobacteria" (1/35/4.82E-7)
» » » class Actinobacteria (1/35/4.82E-7)
» » » » subclass Actinobacteridae (1/31/3.18E-6)
» » » » » order Actinomycetales (1/31/3.18E-6)
» » » » » » suborder Streptosporangineae (0/9/1.36E-2)
» » » » » » » unclassified Streptosporangineae (0/9/NA)
» » » » » » unclassified Actinomycetales (1/22/NA)
» » » » unclassified Actinobacteria (0/4/NA)
» » phylum "Verrucomicrobia" (1/0/3.08E-1)
» » » unclassified "Verrucomicrobia" (1/0/NA)
» » phylum "Firmicutes" (7/30/6.34E-3)
» » » class "Clostridia" (0/5/1E-1)
» » » » order Clostridiales (0/5/1E-1)
» » » » » family "Lachnospiraceae" (0/1/7.38E-1)
» » » » » » unclassified "Lachnospiraceae" (0/1/NA)
» » » » » family Clostridiaceae (0/1/7.38E-1)
» » » » » » subfamily "Clostridiaceae 1" (0/1/7.38E-1)
» » » » » » » unclassified "Clostridiaceae 1" (0/1/NA)
» » » » » family Incertae Sedis XI (0/1/7.38E-1)
» » » » » » genus Tepidimicrobium (0/1/7.38E-1)
» » » » » unclassified Clostridiales (0/2/NA)
» » » class "Bacilli" (5/15/2.15E-1)
» » » » order "Lactobacillales" (1/0/3.08E-1)
» » » » » family "Carnobacteriaceae" (1/0/3.08E-1)
» » » » » » genus Atopostipes (1/0/3.08E-1)
» » » » order Bacillales (3/14/7.5E-2)
» » » » » family Thermoactinomycetaceae (0/3/2.72E-1)
» » » » » » genus Planifilum (0/2/4.48E-1)
» » » » » » unclassified Thermoactinomycetaceae (0/1/NA)
» » » » » family "Paenibacillaceae" (0/2/4.48E-1)
» » » » » » unclassified "Paenibacillaceae" (0/2/NA)
» » » » » family Caryophanaceae (1/0/3.08E-1)
» » » » » » genus Caryophanon (1/0/3.08E-1)
» » » » » family "Staphylococcaceae" (1/0/3.08E-1)
» » » » » » genus Salinicoccus (1/0/3.08E-1)
» » » » » family Bacillaceae (1/7/1.53E-1)
» » » » » » genus Ureibacillus (0/5/1E-1)
» » » » » » genus Geobacillus (0/2/4.48E-1)
» » » » » » unclassified Bacillaceae (1/0/NA)
» » » » » unclassified Bacillales (0/2/NA)
» » » » unclassified "Bacilli" (1/1/NA)
» » » unclassified "Firmicutes" (2/10/NA)
» » phylum "Acidobacteria" (1/1/6.83E-1)
» » » class Acidobacteria_Gp3 (0/1/7.38E-1)
» » » » genus Gp3 (0/1/7.38E-1)
» » » class Acidobacteria_Gp7 (1/0/3.08E-1)
» » » » genus Gp7 (1/0/3.08E-1)
» » phylum "Bacteroidetes" (8/4/5.66E-2)
» » » class "Bacteroidia" (2/0/1.21E-1)
» » » » order "Bacteroidales" (2/0/1.21E-1)
» » » » » family Bacteroidaceae (1/0/3.08E-1)
» » » » » » genus Bacteroides (1/0/3.08E-1)
» » » » » unclassified "Bacteroidales" (1/0/NA)
» » » class Flavobacteria (1/0/3.08E-1)
» » » » order "Flavobacteriales" (1/0/3.08E-1)
» » » » » family Flavobacteriaceae (1/0/3.08E-1)
» » » » » » unclassified Flavobacteriaceae (1/0/NA)
» » » class "Sphingobacteria" (0/3/2.72E-1)
» » » » order "Sphingobacteriales" (0/3/2.72E-1)
» » » » » family "Rhodothermaceae" (0/2/4.48E-1)
» » » » » » genus Rhodothermus (0/2/4.48E-1)
» » » » » family "Chitinophagaceae" (0/1/7.38E-1)
» » » » » » unclassified "Chitinophagaceae" (0/1/NA)
» » » unclassified "Bacteroidetes" (5/1/NA)
» » phylum "Proteobacteria" (82/25/6E-14)
» » » class Alphaproteobacteria (11/18/8.73E-1)
» » » » order Caulobacterales (1/0/3.08E-1)
» » » » » family Caulobacteraceae (1/0/3.08E-1)
» » » » » » unclassified Caulobacteraceae (1/0/NA)
» » » » order Rhizobiales (3/9/3.65E-1)
» » » » » family Methylobacteriaceae (2/0/1.21E-1)
» » » » » » genus Methylobacterium (2/0/1.21E-1)
» » » » » unclassified Rhizobiales (1/9/NA)
» » » » order Sphingomonadales (7/0/1.13E-3)
» » » » » family Sphingomonadaceae (7/0/1.13E-3)
» » » » » » genus Sphingomonas (6/0/2.88E-3)
» » » » » » unclassified Sphingomonadaceae (1/0/NA)
» » » » unclassified Alphaproteobacteria (0/9/NA)
» » » class Gammaproteobacteria (4/4/5.03E-1)
» » » » order Chromatiales (1/0/3.08E-1)
» » » » » unclassified Chromatiales (1/0/NA)
» » » » order Pseudomonadales (2/0/1.21E-1)
» » » » » family Pseudomonadaceae (1/0/3.08E-1)
» » » » » » unclassified Pseudomonadaceae (1/0/NA)
» » » » » family Moraxellaceae (1/0/3.08E-1)
» » » » » » genus Psychrobacter (1/0/3.08E-1)
» » » » unclassified Gammaproteobacteria (1/4/NA)
» » » class Betaproteobacteria (64/0/8.26E-27)
» » » » order Burkholderiales (63/0/2.1E-26)
» » » » » family Comamonadaceae (8/0/4.44E-4)
» » » » » » genus Variovorax (7/0/1.13E-3)
» » » » » » unclassified Comamonadaceae (1/0/NA)
» » » » » family Oxalobacteraceae (37/0/7.52E-16)
» » » » » » genus Massilia (10/0/6.86E-5)
» » » » » » unclassified Oxalobacteraceae (27/0/NA)
» » » » » family Burkholderiaceae (15/0/6.4E-7)
» » » » » » genus Burkholderia (14/0/1.63E-6)
» » » » » » unclassified Burkholderiaceae (1/0/NA)
» » » » » unclassified Burkholderiales (3/0/NA)
» » » » unclassified Betaproteobacteria (1/0/NA)
» » » unclassified "Proteobacteria" (3/3/NA)
» » unclassified Bacteria (8/67/NA)

MICHIGAN STATE UNIVERSITY Advancing Knowledge. Transforming Lives.
© 1992-2009 Michigan State University Board of Trustees, East Lansing MI 48824
MSU is an affirmative-action, equal-opportunity employer.

Library Comparison Summary

[start over | tabular view | help]

Library Compare: RDP Naive Bayesian rRNA Classifier Version 2.2, March 2010
Taxonomical Hierarchy: RDP training set 6, based on nomenclatural taxonomy and Bergey's Manual
Query Submit Date: Fri Dec 17 14:46:54 EST 2010

Library1: *AM8 son aerosols.txt* Total # of sequences: 147
Library2: *AM8 mod aerosols.txt* Total # of sequences: 108

Display depth: 10 **Confidence threshold:** 80% Refresh

Library1	%	phylum	%	Library2
	0.0	"Verrucomicrobia"	0.9	
	0.0	"Acidobacteria"	0.9	
	0.7	"Spirochaetes"	0.0	
	6.8	"Actinobacteria"	0.9	
	13.6	"Bacteroidetes"	7.4	
	34.7	"Firmicutes" *	6.5	
	19.0	"Proteobacteria" *	75.9	
	25.2	unclassified Bacteria	7.4	

(* = significantly different at 0.01)

Lineage ***(click to return to particular node):***

Root (147/108/1.01E0)

Hierarchy View ***(click a node to make it the root -- click the root to see sequence assignment detail):***

domain Bacteria (147/108/1.01E0) (library1/library2/significance value) [*show assignment detail*]
» » phylum "Verrucomicrobia" (0/1/3.59E-1)
» » » class Spartobacteria (0/1/3.59E-1)
» » » » genus Spartobacteria_genera_incertae_sedis (0/1/3.59E-1)
» » phylum "Acidobacteria" (0/1/3.59E-1)
» » » unclassified "Acidobacteria" (0/1/NA)
» » phylum "Spirochaetes" (1/0/6.65E-1)
» » » class Spirochaetes (1/0/6.65E-1)
» » » » order Spirochaetales (1/0/6.65E-1)
» » » » » family Spirochaetaceae (1/0/6.65E-1)
» » » » » » genus Treponema (1/0/6.65E-1)
» » phylum "Actinobacteria" (10/1/2.64E-2)
» » » class Actinobacteria (10/1/2.64E-2)
» » » » subclass Actinobacteridae (10/1/2.64E-2)
» » » » » order Actinomycetales (10/1/2.64E-2)
» » » » » » suborder Propionibacterineae (1/0/6.65E-1)
» » » » » » » family Nocardioidaceae (1/0/6.65E-1)
» » » » » » » » genus Nocardioides (1/0/6.65E-1)
» » » » » » suborder Micrococcineae (2/0/3.83E-1)
» » » » » » » unclassified Micrococcineae (2/0/NA)
» » » » » » suborder Corynebacterineae (2/0/3.83E-1)
» » » » » » » family Corynebacteriaceae (1/0/6.65E-1)
» » » » » » » » genus Corynebacterium (1/0/6.65E-1)
» » » » » » » unclassified Corynebacterineae (1/0/NA)
» » » » » » unclassified Actinomycetales (5/1/NA)
» » phylum "Bacteroidetes" (20/8/1.19E-1)
» » » class "Sphingobacteria" (0/1/3.59E-1)
» » » » order "Sphingobacteriales" (0/1/3.59E-1)
» » » » » unclassified "Sphingobacteriales" (0/1/NA)
» » » class Flavobacteria (4/1/3.97E-1)
» » » » order "Flavobacteriales" (4/1/3.97E-1)
» » » » » family Flavobacteriaceae (2/1/8.7E-1)
» » » » » » unclassified Flavobacteriaceae (2/1/NA)
» » » » » unclassified "Flavobacteriales" (2/0/NA)
» » » class "Bacteroidia" (8/2/1.81E-1)
» » » » order "Bacteroidales" (8/2/1.81E-1)
» » » » » family "Porphyromonadaceae" (1/0/6.65E-1)
» » » » » » unclassified "Porphyromonadaceae" (1/0/NA)
» » » » » unclassified "Bacteroidales" (7/2/NA)
» » » unclassified "Bacteroidetes" (8/4/NA)
» » phylum "Firmicutes" (51/7/1.16E-7)
» » » class "Bacilli" (7/6/7.79E-1)
» » » » order "Lactobacillales" (0/1/3.59E-1)
» » » » » family "Carnobacteriaceae" (0/1/3.59E-1)
» » » » » » genus Atopostipes (0/1/3.59E-1)
» » » » order Bacillales (6/3/6.5E-1)
» » » » » family Bacillaceae (0/1/3.59E-1)
» » » » » » unclassified Bacillaceae (0/1/NA)
» » » » » family Caryophanaceae (1/1/7.72E-1)
» » » » » » genus Caryophanon (1/1/7.72E-1)
» » » » » family "Staphylococcaceae" (3/1/5.95E-1)
» » » » » » genus Salinicoccus (0/1/3.59E-1)
» » » » » » genus Staphylococcus (1/0/6.65E-1)
» » » » » » genus Jeotgalicoccus (2/0/3.83E-1)
» » » » » unclassified Bacillales (2/0/NA)
» » » » unclassified "Bacilli" (1/2/NA)
» » » class "Erysipelotrichi" (2/0/3.83E-1)
» » » » order "Erysipelotrichales" (2/0/3.83E-1)
» » » » » family Erysipelotrichaceae (2/0/3.83E-1)
» » » » » » genus Turicibacter (2/0/3.83E-1)
» » » class "Clostridia" (42/0/1.03E-10)
» » » » order Clostridiales (36/0/2.82E-9)
» » » » » family Veillonellaceae (2/0/3.83E-1)
» » » » » » unclassified Veillonellaceae (2/0/NA)
» » » » » family Clostridiaceae (1/0/6.65E-1)
» » » » » » subfamily "Clostridiaceae 1" (1/0/6.65E-1)
» » » » » » » unclassified "Clostridiaceae 1" (1/0/NA)
» » » » » family "Lachnospiraceae" (13/0/8.95E-4)
» » » » » » genus Butyrivibrio (1/0/6.65E-1)
» » » » » » unclassified "Lachnospiraceae" (12/0/NA)
» » » » » family "Ruminococcaceae" (8/0/1.41E-2)
» » » » » » genus Oscillibacter (1/0/6.65E-1)
» » » » » » unclassified "Ruminococcaceae" (7/0/NA)
» » » » » unclassified Clostridiales (12/0/NA)
» » » » unclassified "Clostridia" (6/0/NA)
» » » unclassified "Firmicutes" (0/1/NA)
» » phylum "Proteobacteria" (28/82/6E-14)
» » » class Alphaproteobacteria (7/11/9.49E-2)
» » » » order Caulobacterales (0/1/3.59E-1)
» » » » » family Caulobacteraceae (0/1/3.59E-1)
» » » » » » unclassified Caulobacteraceae (0/1/NA)
» » » » order Rhizobiales (5/3/8.49E-1)
» » » » » family Methylobacteriaceae (0/2/1.52E-1)
» » » » » » genus Methylobacterium (0/2/1.52E-1)
» » » » » unclassified Rhizobiales (5/1/NA)
» » » » order Rhodospirillales (1/0/6.65E-1)
» » » » » family Rhodospirillaceae (1/0/6.65E-1)
» » » » » » genus Azospirillum (1/0/6.65E-1)
» » » » order Sphingomonadales (1/7/1.16E-2)
» » » » » family Sphingomonadaceae (1/6/2.46E-2)
» » » » » » genus Sphingomonas (1/6/2.46E-2)
» » » » » unclassified Sphingomonadales (0/1/NA)
» » » class Betaproteobacteria (10/63/6E-14)
» » » » order Burkholderiales (10/62/6E-14)
» » » » » family Alcaligenaceae (1/0/6.65E-1)
» » » » » » genus Sutterella (1/0/6.65E-1)
» » » » » family Oxalobacteraceae (3/36/6.28E-11)
» » » » » » genus Massilia (0/9/3.71E-4)
» » » » » » unclassified Oxalobacteraceae (3/27/NA)
» » » » » family Burkholderiaceae (5/15/3.49E-3)
» » » » » » genus Burkholderia (3/14/9.1E-4)
» » » » » » unclassified Burkholderiaceae (2/1/NA)
» » » » » family Comamonadaceae (1/8/5.43E-3)
» » » » » » genus Variovorax (0/7/2.07E-3)
» » » » » » unclassified Comamonadaceae (1/1/NA)
» » » » » unclassified Burkholderiales (0/3/NA)
» » » » unclassified Betaproteobacteria (0/1/NA)
» » » class Gammaproteobacteria (11/4/2.47E-1)
» » » » order Chromatiales (0/1/3.59E-1)
» » » » » unclassified Chromatiales (0/1/NA)
» » » » order Xanthomonadales (2/0/3.83E-1)
» » » » » family Xanthomonadaceae (2/0/3.83E-1)
» » » » » » unclassified Xanthomonadaceae (2/0/NA)
» » » » order "Enterobacteriales" (2/0/3.83E-1)
» » » » » family Enterobacteriaceae (2/0/3.83E-1)
» » » » » » genus Escherichia/Shigella (1/0/6.65E-1)
» » » » » » unclassified Enterobacteriaceae (1/0/NA)
» » » » order Pseudomonadales (2/2/7.18E-1)
» » » » » family Moraxellaceae (0/1/3.59E-1)
» » » » » » genus Psychrobacter (0/1/3.59E-1)
» » » » » family Pseudomonadaceae (1/1/7.72E-1)
» » » » » » genus Pseudomonas (1/0/6.65E-1)
» » » » » » unclassified Pseudomonadaceae (0/1/NA)
» » » » » unclassified Pseudomonadales (1/0/NA)
» » » » unclassified Gammaproteobacteria (5/1/NA)
» » » unclassified "Proteobacteria" (0/4/NA)
» » unclassified Bacteria (37/8/NA)

© 1992-2009 Michigan State University Board of Trustees, East Lansing MI 48824
MSU is an affirmative-action, equal-opportunity employer.
